# Supplementary material for: Silver Nanoparticle Sensor Array-Based Meat Freshness Inspection System
Source: Foods. 2023 Oct 18;12(20):3814. doi: 10.3390/foods12203814 (PMC10606817; doi:10.3390/foods12203814)
Supplement: Supplementary file 1 [file foods-12-03814-s001.zip › foods-2660497-SI.pdf]

Supporting Information

Silver Nanoparticle Sensor Array-Based Meat Freshness Inspection System

Jiahang Yu [1,2], Mingyuan Huang [1], Huixin Tian [1] and Xinglian Xu [1,*]

[1] State Key Laboratory of Meat Quality Control and Cultured Meat Development, Jiangsu Collaborative Innovation Center of Meat Production and Processing, Quality and Safety Control, College of Food Science and technology, Nanjing Agricultural University, Nanjing 210095, China; yjh8319@yeah.net (J.Y.); hnzzhmy@foxmail.com (M.H.); yjh@chzu.edu.cn (H.T.)

[2] School of Biological Science and Food Engineering, Chuzhou University, Chuzhou 239000, China

* Correspondence: xlxus@njau.edu.cn; Tel.: +86-025-84395939; Fax: +86-025-84395730

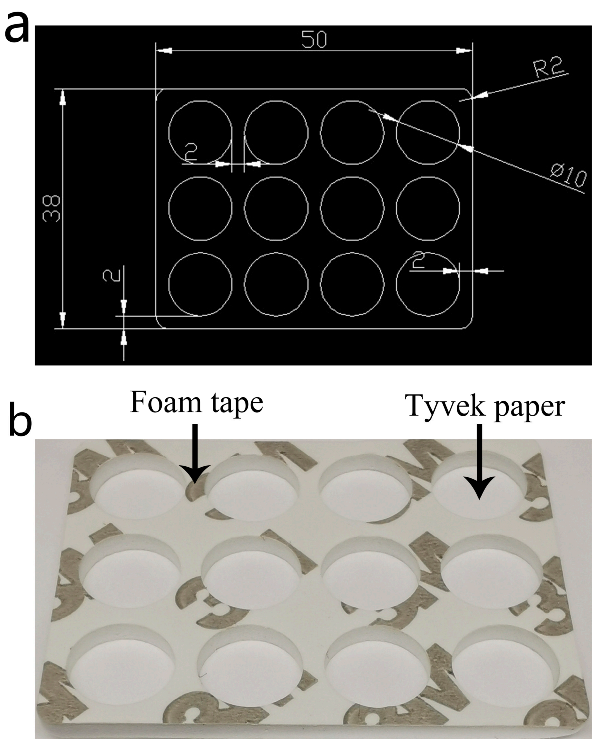

Figure S1. Preparation of colorimetric gel tank: (a) CAD top-side view; (b) entity graph; made from foam tape with a 10 mm inner ring radius. The bottom is sealed with Tyvek paper, which has excellent breathability and safety.

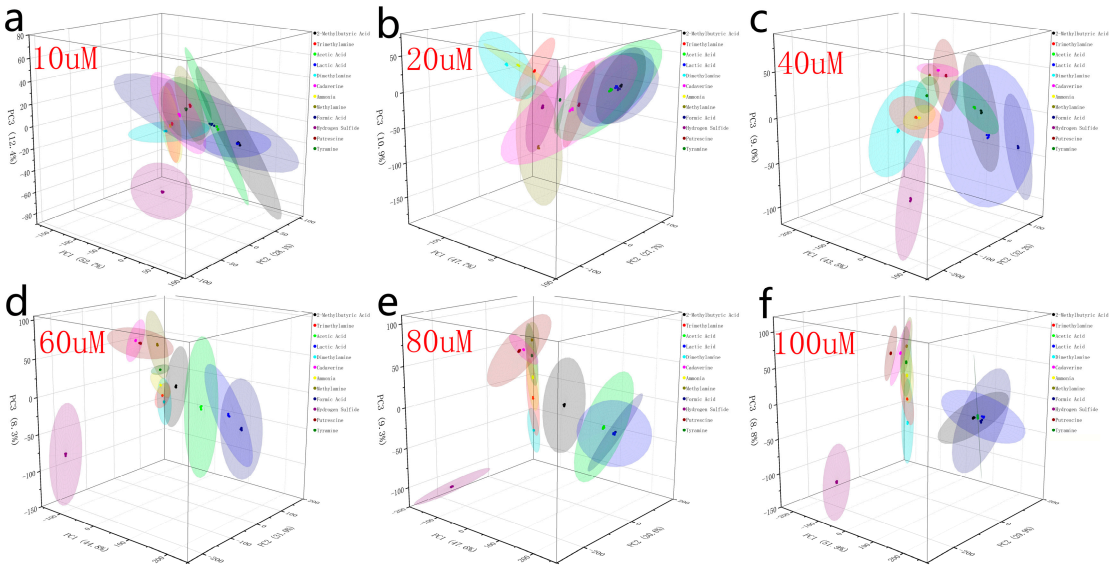

Figure S2. Clusters of twelve VOCs via 3D PCA score plot.

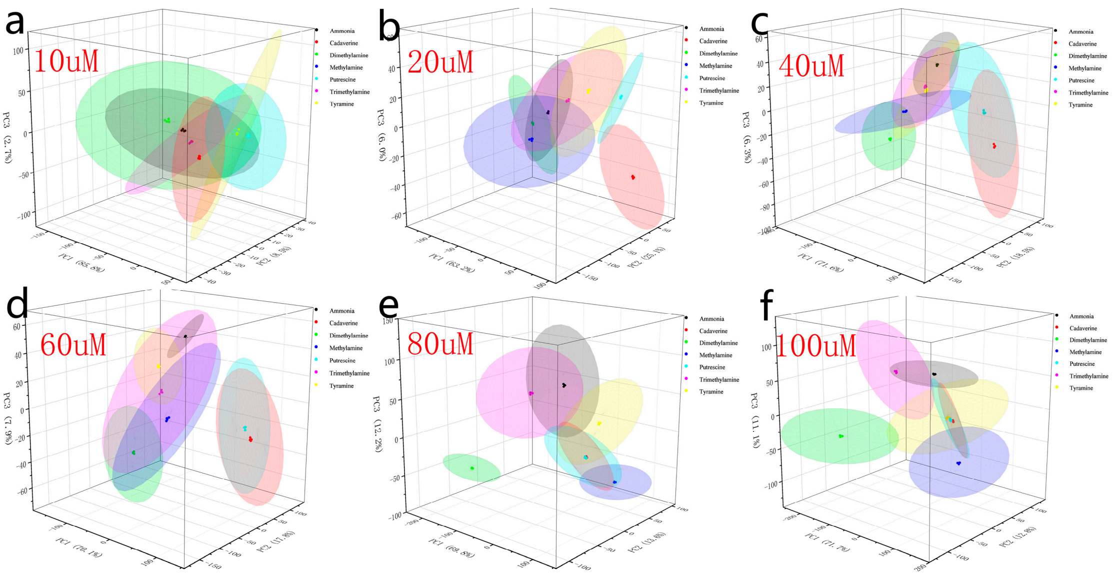

Figure S3. Clusters of seven amines via 3D PCA score plot.

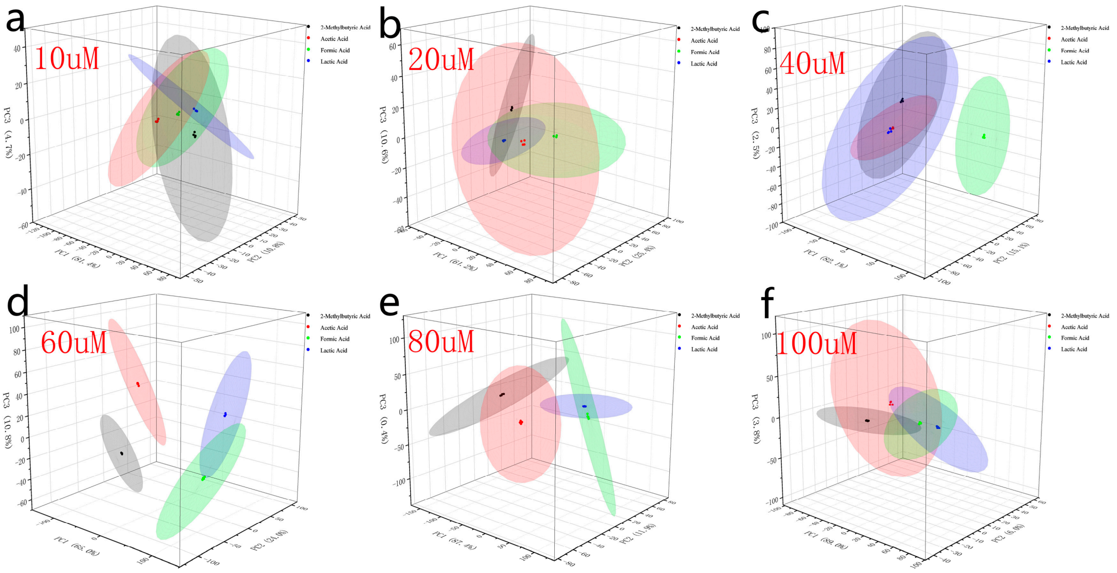

Figure S4. Clusters of four acids via 3D PCA score plot.

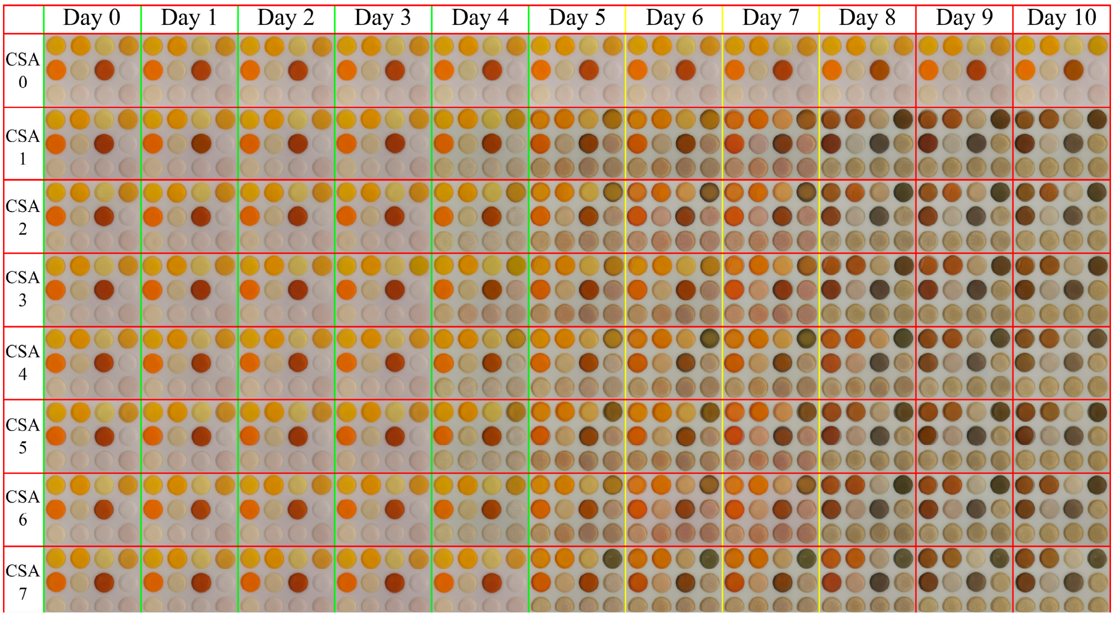

Figure S5. Color changes of CSA in chilled broiler meat wrapped in modified atmosphere packaging (MAP: 20% CO_2, 80% N_2) at 4 °C. (Eight data sets in total)

REFERENCES

[43] Zhang, X., Wang, H., Li, N., Li, M., Xu, X., High CO2-modified atmosphere packaging for extension of shelf-life of chilled yellow-feather broiler meat: A special breed in Asia. LWT–Food Sci. Technol. 2015, 64, 1123-1129.
